# Respiratory pandemic preparedness learnings from the June 2020 COVID-19 outbreak at San Quentin California State Prison

**DOI:** 10.1108/IJPH-12-2021-0116

**Published:** 2022-06-10

**Authors:** Ada Kwan, Rachel Sklar, Drew B. Cameron, Robert C. Schell, Stefano M. Bertozzi, Sandra I. McCoy, Brie Williams, David A. Sears

**Affiliations:** Division of Pulmonary and Critical Care Medicine, Department of Medicine, University of California San Francisco, San Francisco, California, USA and Division of Health Policy and Management, School of Public Health, University of California, Berkeley, Berkeley, California, USA.; Program on Reproductive Health and the Environment, University of California San Francisco, San Francisco, California, USA.; Division of Health Policy and Management, School of Public Health, University of California, Berkeley, Berkeley, California, USA and Department of Health Policy and Management, Yale School of Public Health, Yale University, New Haven, Connecticut, USA.; Division of Health Policy and Management, School of Public Health, University of California, Berkeley, Berkeley, California, USA.; Division of Health Policy and Management, School of Public Health, University of California, Berkeley, Berkeley, California, USA; School of Public Health, University of Washington, Seattle, Washington, USA and Instituto Nacional de Salud Pública, Cuernavaca, Mexico.; Division of Epidemiology, School of Public Health, University of California, Berkeley, Berkeley, California, USA.; Center for Vulnerable Populations, Department of Medicine, University of California San Francisco, San Francisco, California, USA.; Division of Infectious Diseases, Department of Medicine, University of California San Francisco, San Francisco, California, USA.

**Keywords:** COVID-19, Prison health, Decarceration, Outbreak investigation, Pandemic preparedness, Prison environment, SARS-CoV-2, Ventilation, Prisons

## Abstract

**Purpose –:**

This study aims to characterize the June 2020 COVID-19 outbreak at San Quentin California State Prison and to describe what made San Quentin so vulnerable to uncontrolled transmission.

**Design/methodology/approach –:**

Since its onset, the COVID-19 pandemic has exposed and exacerbated the profound health harms of carceral settings, such that nearly half of state prisons reported COVID-19 infection rates that were four or more times (and up to 15 times) the rate found in the state’s general population. Thus, addressing the public health crises and inequities of carceral settings during a respiratory pandemic requires analyzing the myriad factors shaping them. In this study, we reported observations and findings from environmental risk assessments during visits to San Quentin California State Prison. We complemented our assessments with analyses of administrative data.

**Findings –:**

For future respiratory pathogens that cannot be prevented with effective vaccines, this study argues that outbreaks will no doubt occur again without robust implementation of additional levels of preparedness – improved ventilation, air filtration, decarceration with emergency evacuation planning – alongside addressing the vulnerabilities of carceral settings themselves.

**Originality/value –:**

This study addresses two critical aspects that are insufficiently covered in the literature: how to prepare processes to safely implement emergency epidemic measures when needed, such as potential evacuation, and how to address unique challenges throughout an evolving pandemic for each carceral setting.

## Introduction

The coronavirus disease 2019 (COVID-19) pandemic has exposed the profound public health dangers posed by carceral settings, which imprison some of society’s most medically marginalized people (Bick, 2007; Moazen *et al.*, 2019; Brennan, 2020; Lemasters *et al.*, 2020; Muntingh, 2020; Rapisarda *et al.*, 2020; Reinhart and Chen, 2020; Saloner *et al.*, 2020; Barnert *et al.*, 2021; Cingolani *et al.*, 2021). In the USA, which holds a quarter of the world’s incarcerated population, nearly half of state prisons reported COVID-19 infection rates that were four or more times – and up to 15 times – the rate found in each state’s general population; COVID-19 deaths among incarcerated people have been three times the general population; and among staff, reported infection rates have been three times the general population (National Academies of Sciences, Engineering and Medicine, 2021; New York Times, 2021). As we pass two years since the onset of the pandemic, we must better understand the factors that make carceral settings so health-harming alongside the failures in COVID-19 control strategies that have permitted the emergence of striking health disparities between people who are and are not incarcerated (Duarte *et al.*, 2022).

The scientific literature has documented what makes carceral settings dangerous, characterized outbreaks associated with the severe acute respiratory syndrome coronavirus 2 (SARS-CoV-2, the virus that causes COVID-19) in carceral settings and identified priorities to address COVID-19 in these settings, notably population reduction, ventilation, appropriate mask use, quarantine, medical isolation and high vaccination rates once effective vaccines became available (Barnert *et al.*, 2021; Barsky *et al.*, 2021; National Academies of Sciences, Engineering and Medicine, 2021). This article contributes to the literature by characterizing the large COVID-19 outbreak at San Quentin State Prison in northern California, identifying San Quentin’s particular vulnerabilities and deconstructing key measures that must be robustly implemented to prevent such a widespread outbreak from occurring again (Kothari *et al.*, 2020; Sears *et al.*, 2020). Further, we discuss two critical aspects insufficiently addressed in the literature: how a carceral setting must be prepared to safely implement emergency epidemic measures, including evacuation and how implementing mitigation measures throughout an evolving pandemic involves unique challenges for each carceral setting.

On June 13, 2020, two weeks after 122 incarcerated people were transferred (with an undetermined number having COVID-19) to San Quentin, members of our multidisciplinary team of clinicians, epidemiologists, exposure scientists and health policy researchers visited the prison to evaluate the risk of an uncontrolled outbreak. We went at the request of the Federal Receivership which oversees healthcare in California state prisons. Over the course of the pandemic, the California Department of Corrections and Rehabilitation (CDCR), with the second largest state prison population in the USA, has experienced a devastating series of COVID-19 outbreaks throughout its 35 prisons, without a single facility spared. The San Quentin outbreak was an extreme example of an explosive outbreak that spread to 2,241 (62.3%) incarcerated people and 445 (27%) staff during the outbreak.

On the morning of our visit, the institution had approximately 20 confirmed active cases. Two days later, we issued an urgent memo (Appendix A) describing risks for rapid and widespread SARS-CoV-2 transmission at San Quentin and urgent recommendations to mitigate these risks (McCoy *et al.*, 2020). What we witnessed was the outset of a SARS-CoV-2 outbreak that swiftly became one of the nation’s largest and most publicized (Maxmen, 2020; New York Times, 2020, 2021). In this paper, we draw on our observations from visits to the institution and an examination of publicly available and administrative data to argue how robust implementation of widely recognized mitigation measures is vital to protecting lives and preventing outbreaks in the future (Barnert *et al.*, 2021; Barsky *et al.*, 2021; National Academies of Sciences, Engineering and Medicine, 2021).

## Data sources and ethics approval

Some data presented in this paper include summaries of de-identified administrative and health records ranging from January 1, 2019, to July 17, 2021, which were provided to the authors by the CDCR and California Correctional Health Care Services (CCHCS). The University of California (UC), San Francisco Human Research Protection Program Institutional Review Board (IRB) (IRB#: 21–34030) has approved the use of this data for research under 45 CFR 305(a), and the data use protocol is also registered in the UC Berkeley IRB Reliance Registry (Study #3755).

Other data presented in this paper are derived from observations during an on-site assessment conducted on June 13, 2020, at San Quentin State Prison in California, as well as subsequent visits to the institution in 2021. The assessment and subsequent visits were requested by the office of the California Prison Health Care Receivership to assess the risks within the environment for SARS-CoV-2 transmission. Our on-site assessment and use of publicly available data did not involve experimental protocols. Using their self-assessment tool, the UC Berkeley Committee for the Protections of Human Subjects determined that under Federal Regulations 21 CFR 50.3 and 45 CFR 46.102, our quality improvement activities were not deemed “human subjects research” and written, informed consent was not required for this assessment. All methods were carried out in accordance with relevant guidelines and regulations.

## Characterization of San Quentin’s COVID-19 outbreak

In early 2020, the California Institute for Men (CIM), another CDCR prison, was experiencing a rapidly evolving, facility-wide COVID-19 outbreak. With increasingly limited options to minimize transmission risk, CIM leaders assessed and identified people at increased risk for adverse COVID-19 outcomes (primarily from dormitories) for transfer to cells at San Quentin. At the time, San Quentin’s population was at 114% of architectural design capacity, a decrease from 135% in January 2020.

**Figure 1:**
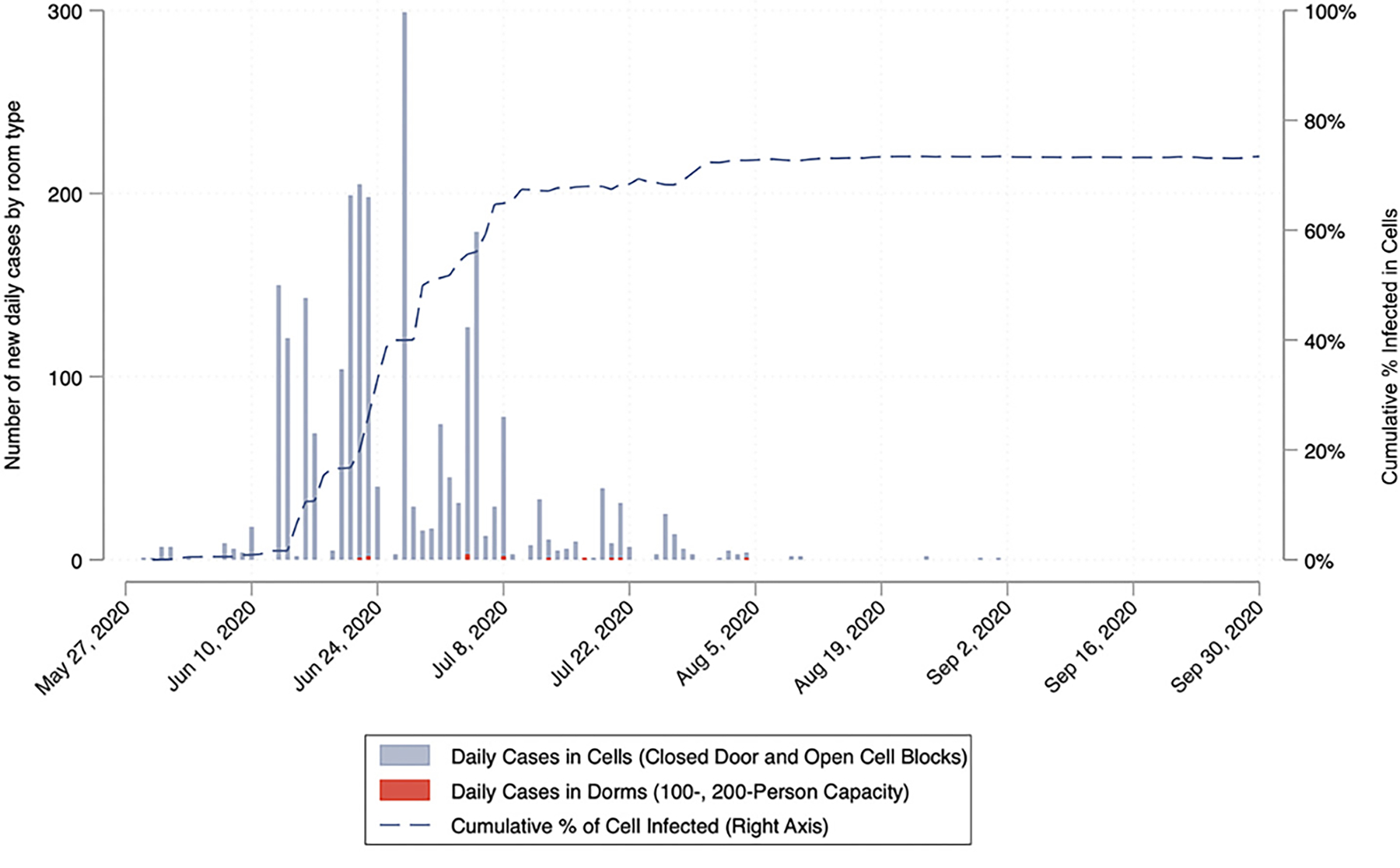
New daily COVID-19 cases by room type over time at San Quentin **Notes:** During the San Quentin outbreak, COVID transmission spread quickly through the closed dormitory cellblocks of North, South, East and West blocks (where 85% of incarcerated people resided) and was kept out of the two 100-person and three 200-person dormitories (where 14% of incarcerated people resided). Over the course of the pandemic, a large proportion of people were infected, and staff moved individuals to various buildings in the institution for quarantine and medical isolation

On May 30, 2020, 122 incarcerated people from CIM boarded five buses to San Quentin, which had yet to have any known COVID-19 infections (Office of the Inspector General, 2021). Upon arrival, two men reported COVID-like symptoms. Figure 1 depicts the number of cases over the course of the outbreak. Within 22 days post-transfer, the number of active cases among incarcerated people at San Quentin increased to 500, far exceeding the number of solid-door cells for safe medical isolation. Five days later, active cases more than doubled to 1,198. At 38 days, daily active cases peaked at 1,635 and returned to zero on September 25, 2020. Between May 30 and September 30, there were 2,268 total confirmed cases (62.3% of the 3,643 total population ever present during the outbreak) and 28 deaths among incarcerated people, with the true attack rate even higher because many did not consent to be tested. Overall, COVID-19 spread rapidly through the cellblocks, where 85% of the incarcerated people resided. It largely spared the dormitories located in a separate yard where 15% of the San Quentin population resided. Among staff, there were 445 (27% of 1,678) reported cases and one death.

## Vulnerabilities that contributed to San Quentin’s outbreak

Once SARS-CoV-2 entered San Quentin, few preventive actions recommended for containing community spread could be implemented to slow the rapid pace of transmission. This problem was not new but was described a century ago when San Quentin experienced three 1918 influenza outbreaks (Stanley, 1919; Hawks *et al.*, 2020; Franco-Paredes *et al.*, 2021). Resonant of recommendations from that period, the following vulnerabilities contributed to San Quentin’s COVID-19 outbreak:

### Environmental risks enabled airborne transmission

San Quentin is California’s oldest state prison, with buildings from the mid-1800s and early 1900s. A lack of rooms that have isolated airspace from others – coupled with overcrowding – created a superspreading environment during the 1918 influenza and the COVID-19 pandemics. Design elements of residential units, particularly the barred and perforated metal doors, allow for open air exchange and enable rapid diffusion of aerosols within spaces housing up to 800 people.

Among the 152 cells in the entire prison with solid walls and solid doors, 50 are in the medical building and 102 are in the “Adjustment Center”– a location otherwise used for punitive and restricted confinement (i.e. “solitary confinement” and death row). Despite the fact that using cells normally used for punishment for quarantine has been associated with documented health harms (Williams, 2016; Haney *et al.*, 2020), people who arrived from CIM with symptoms were placed in the Adjustment Center, which had approximately 16 unoccupied closed-door cells at the time.

Those who arrived without symptoms were placed singly in four-by-ten-foot cells primarily on the top tiers of “the Badger Unit.” The 494-capacity Badger unit is one of four units located in one of four cellblocks referred to as “closed dormitories”, each with five floors of cells. These cells (3,851 capacity in total) have solid floors, solid ceilings and solid walls on three sides. Nearly, all the cells have bars or perforated metal on the fourth side, which faces an open atrium spanning all floors. Because air circulates freely out of one cell and into another, each cellblock functions more like a dormitory rather than a facility with isolated prison cells.

Further, San Quentin’s cellblocks were originally built with large windows to provide cross-ventilation, but in recent years those have been welded shut, preventing natural ventilation for the buildings as originally designed. Fresh air is introduced to the cellblocks via a mechanical system that brings fresh air onto the first floor. A circulating fan on the fifth floor circulates air within each building. The circulation and mixing of air ensure that fresh air and, in the wintertime, heated air are distributed to each floor in the five-floor cellblock. This mixing mechanism may also serve to transport infectious viruses from one area to another, especially because there is no solid door separating infected individuals from uninfected individuals and common areas.

While circulated air is passed through a filter on circulation fans, filters were low-grade pleated filters not rated for viral particle removal (ASHRAE, 2021). Given the overall configuration of the cellblocks and the air circulation, aerosols emitted from infected individuals may have freely diffused from an infected cell to common areas, other nearby cells through the perforated metal door and the rest of the building, where staff worked across three shifts each day.

### Human resources in short supply

Managing the outbreak required additional human resources to provide urgent medical care to the large numbers of people who were sick and testing positive for SARS-CoV-2, make quarantine and medical isolation decisions, liaise with community hospitals, address environmental risk factors (e.g. poorly functioning vents, enhancing natural ventilation) and ensure the safe movement and security of incarcerated people and staff. Absence of previously developed epidemic response plans (e.g. ventilation response plans, isolation/quarantine plans) further stressed staff capacity as staff were required to develop plans during the emergency, while putting aside their existing responsibilities.

### Delays in testing turnaround

**Figure 2:**
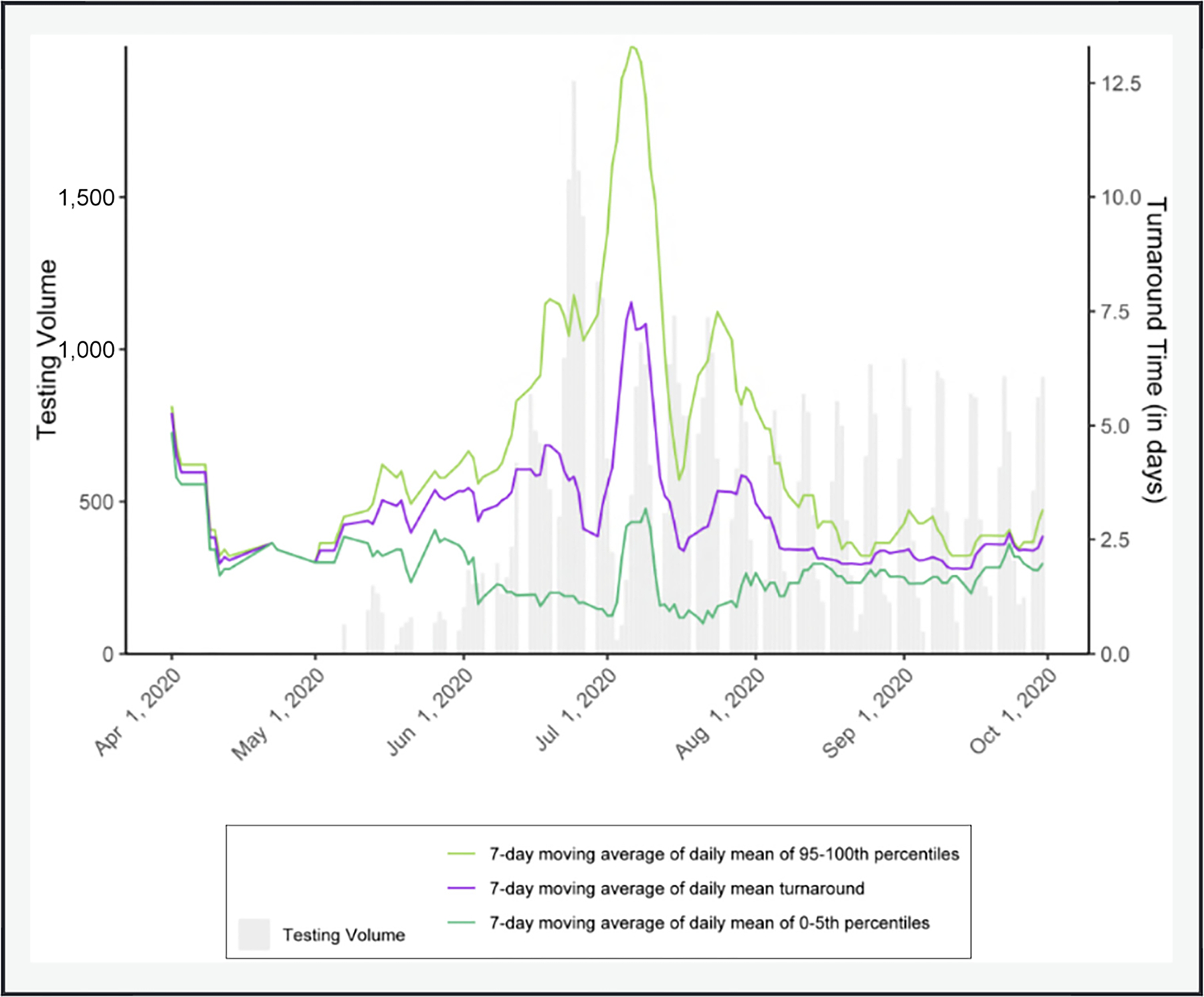
In addition to being over 100% design capacity, testing turnaround time at San Quentin during the outbreak made it challenging to make timely quarantine and isolation decisions

Timely test results facilitate decisions about control measures like medical isolation and quarantine to prevent onward spread of SARS-CoV-2. Figure 2 shows testing turnaround time averaged 7.5 days during San Quentin’s outbreak. Given that the average duration of SARS-CoV-2 infectiousness is similar to that turnaround time, the great majority of transmission had already occurred while staff awaited results to make quarantine and isolation decisions (He *et al.*, 2020; McAloon *et al.*, 2020; Wang *et al.*, 2020; Williams *et al.*, 2020; Cevik *et al.*, 2021).

It is important to underscore the relationship between the built environment and testing delays. While we do not believe that it is possible to avoid large outbreaks in settings like San Quentin based on current design, we also do not know how much the attack rate could have been reduced had rapid-turnaround testing been available and had the environmental controls identified been immediately implemented. When COVID-19 was introduced into a San Quentin cellblock, there was no more urgent need for rapid-turnaround testing in the state (McCoy *et al.*, 2020). In a similar situation in the future, the state public health department should prioritize testing to ensure rapid turnaround, which is essential for reducing ongoing transmission when it is not possible to remove exposed persons from a high-risk congregate environment.

### Insufficient quarantine and isolation space

Ideally, a prison can address an outbreak in a highly vulnerable setting by moving infected and exposed people to individual isolation and quarantine. Even disregarding the fact that only 16 individual, closed-door cells were unoccupied at the beginning of San Quentin’s outbreak, only having 152 beds in solid door celled housing in the entire institution means that leadership cannot move the population that resides in even one of its dormitories or cellblock units into safe quarantine. Because 102 of those cells are normally used for punitive solitary confinement and death row, even if there were very clear understanding that movement would not involve loss of privileges or delays in receiving belongings, incarcerated people may want to avoid such a move, creating incentives to conceal symptoms or refuse testing (Cloud *et al.*, 2020; Williams *et al.*, 2020).

### A high health-risk population

People incarcerated in US prisons and staff experience higher health condition-related burdens than their age-matched counterparts outside of prison (Maruschak *et al.*, 2015). These include conditions (e.g. diabetes, chronic obstructive pulmonary disease) that put an individual at increased risk for severe COVID-19 outcomes and treatment needs (e.g. dialysis) that can have dire consequences if interrupted. People incarcerated at San Quentin were at even higher medical risk when compared to the overall CDCR population (see Table 1). For example, just 19% of the incarcerated population across CDCR was 55 years or older compared to 39% of people incarcerated at San Quentin. In addition, the average COVID-19 risk score (a score constructed by CDCR that sums weighted values assigned to healthcare conditions for any given incarcerated person) at San Quentin was significantly higher than the average for CDCR overall (see Appendix B for details on the risk score).

### Unwillingness to strictly restrict movement from infected to uninfected housing units

Once infection is detected within an institution, incarcerated people and staff working in an infected housing unit must be considered exposed and prevented from moving to other areas while they might be infectious. A basic principle of infectious disease management is the concept of preventing spread of infection from one population to a neighboring population. Additionally influenced by safety considerations, operational necessity, resource limitations and other processes set forth in the union contract, the pandemic was not enough of an emergency to warrant overriding the state’s contract with the union that allows correctional officers to bid on overtime work by seniority. Thus, correctional officers could work first shift in an infected housing unit and days later work in different, uninfected housing units without being tested on their second shift–even if the only reason for that cross-over was an individual’s preference for overtime. Although San Quentin’s outbreak was caused by a transfer of infected incarcerated people (which differs from many major outbreaks that have been linked to staff introductions), such a lack of staff separation in units with active infections likely interfered with the goal of containing an outbreak to a single housing unit.

Together, these vulnerabilities created an environment which required extreme and additional levels of mitigation. Superspreader events have tended to involve crowds, singing or shouting, poorly ventilated spaces and a lack of adequate face coverings (Lu *et al.*, 2020; McMichael *et al.*, 2020; Miller *et al.*, 2020; Park *et al.*, 2020). These events shared commonalities with what our team observed during our evaluation of San Quentin in June 2020, apart from one factor: the staff and those incarcerated at San Quentin did not attend a superspreader event. Instead, they were contained for long periods of time in a superspreading, biological, social and built environment with the staff entering and exiting daily, returning to communities across northern California.

Ultimately, there were too many people sharing air in poorly ventilated cellblocks for masks, lockdowns including visitation and programming suspension, disinfection and testing to be effective at containing spread. Once SARS-CoV-2 entered, a large outbreak was inevitable and could only be slowed with immediate, rapid decarceration and with much improved ventilation. The alternative – quarantining in place while awaiting test results – failed to slow SARS-CoV-2 transmission.

## Key measures to avert similar outcomes in Carceral settings

Noting ongoing developments and the growing scientific evidence around SARS-CoV-2 transmission, the findings from our initial assessment remain: averting San Quentin’s outbreak would have required rapid mass decarceration. It is important to note that this paper focuses on the lessons from San Quentin’s outbreak which occurred more than six months before vaccines became available. While vaccination can be effective at controlling an epidemic (and has led to a significant reduction in COVID-19 cases in CDCR), this manuscript is focused on outbreak control lessons for when vaccines are unavailable or not highly protective.

Given that SARS-CoV-2 is highly contagious through aerosols, which was not recognized by many national public health authorities at the time of the outbreak, a similar situation at San Quentin in the future would warrant an unprecedented emergency evacuation in a matter of days to prevent the rapid transmission observed over 2–3 weeks. In this section, we deconstruct in greater detail two key measures that were not adequately anticipated at San Quentin.

## Ventilation and filtration

As SARS-CoV-2 is transmitted from infectious individuals through droplets and aerosols, appropriate building ventilation and filtration systems are important in controlling indoor aerosol transmission risks (Allen and Marr, 2020; Morawska and Cao, 2020). It has become increasingly clear that airborne transmission is the principal mode of SARS-CoV-2 transmission (Greenhalgh *et al.*, 2021). Adding to the risk introduced by the building design itself, we observed activities, such as yelling between cells and exercising indoors. Such activities are known to increase the rate of viral emission from an infected individual and contribute to higher concentrations of viral particles in the shared air and a higher risk of transmission in the space (Barreda *et al.*, 2020; Buonanno, Stabile and Morawska, 2020). Prison “lockdowns” which entail keeping people for long durations indoors can actually increase transmission risk by allowing the accumulation of potentially infectious aerosols in housing units and by moving high-risk activities indoors. Despite the widespread scientific evidence that the virus was primarily transmitted through the airborne route, the lack of broad acknowledgement early in the pandemic by authoritative sources such as the CDC and WHO, made it harder to justify measures to prevent aerosol transmission. In June 2020, CDC and WHO were still recommending that transmission could largely be prevented by separating prisoners by six or more feet and disinfecting common areas, such as showers and telephones (Centers for Disease Control and Prevention, 2021).

The lack of sufficient appreciation of the role of aerosols in SARS-CoV-2 transmission also reduced the perceived urgency of implementing well-known controls for preventing rapid spread of a respiratory pathogen in an indoor congregate space (Nishiura *et al.*, 2020). The lack of existing plans delayed implementation of effective controls: minimizing the number of occupants sharing the same indoor airspace, increasing (fresh) air exchange rates (natural and mechanical), avoiding air recirculation, ensuring adequate filtration and implementing air disinfection in areas where ventilation is difficult to improve; as well as controls implemented in other congregate settings: improving masking compliance, limiting high-emission activities to the outdoors, maximizing time spent outdoors, ventilating during reduced occupancy times and using single-zone air filters to remove virus from the air in congregate areas (Schoen *et al.*, 2014; Almilaji and Thomas, 2020; Beggs and Avital, 2020; Morawska *et al.*, 2020; Mousavi *et al.*, 2020; ASHRAE, 2021; Cheng *et al.*, 2021; Lindsley *et al.*, 2021). However, despite the mitigation recommendations described above, housing such a high number and high density of individuals in buildings creates a scenario where slowing the spread of a respiratory pathogen is nearly impossible (Urrego *et al.*, 2015). Future research should focus on documenting conditions in housing units, as well as analyzing how such factors influence health outcomes and how implementing improved ventilation and filtration strategies can prevent the spread of infectious respiratory pathogens.

## Decarceration

To understand the role of decarceration, it is useful to think of how safe incarcerated persons are from infectious diseases given the physical environment and occupancy of a prison under three different scenarios:

- with no respiratory pandemic;
- with a respiratory pandemic but without an active outbreak in the institution; and
- in a respiratory pandemic with an active outbreak.

However, first, while we do not fully address the many physical and mental health harms associated with incarceration, we must acknowledge the health ramifications for the wellbeing of people currently incarcerated and their families, even in the absence of a pandemic (Sutton and Pacino, 2021). We also recognize that respiratory pandemic planning and response is occurring in the context of historical and contemporary forces that created, maintain and facilitate the expansion of mass incarceration, as well as inequality in who is targeted for mass incarceration, with implications for population health and health inequity (National Academies of Sciences, Engineering and Medicine, 2021).

In the absence of a respiratory pandemic, public health guidelines recommend against 800 people living together in a single, shared airspace. Despite how occupancy levels are deemed necessary by official codes that protect the general safety and welfare of occupants and the public, there is no consistent public health guidance regarding maximum residential room occupancy and no consistent measure for objective prison crowding exists (Simpson *et al.*, 2019). Examples from municipal codes for shelters typically have maximum capacities less than 50 (California Legislative Information, 2020). We argue that very large, “closed dormitories,” such as 800-bed units and other dormitories at San Quentin, are inherently unsafe if over 100 people are spending the majority of their time in a shared, poorly ventilated airspace with multiple staff.

When a respiratory epidemic or pandemic occurs, public health authorities limit indoor gathering to small groups of six or ten maximum. Thus, when a serious threat of a respiratory virus exists, ensuring that people in prison are not living in large groups where transmission can occur rapidly or uncontrollably is pertinent for public health and preventing the overburdening local health systems. As plans exist for evacuation in the event of a wildfire, earthquake or chemical spill, plans must exist for emergency evacuation of high-risk housing units that cannot be made significantly safer during an infectious disease outbreak. In some places, it may be possible to achieve this with temporary housing units (e.g. trailers, tents). Emergency decarceration is a costly and potentially dangerous activity; however, if an evaluation of whether someone can be safely decarcerated is only done after an outbreak has occurred, then it will likely take too long to make that assessment and achieve decarceration quickly enough.

In any high-risk setting, there must be prior assessments (e.g. as soon as a pandemic is declared) of who could be decarcerated into: the community (e.g. to family), an unsecure community setting (e.g. an unsecured hotel), a low-security alternative facility (e.g. a hotel with correctional officers) and who would either need to remain in the facility or be transferred to another correctional facility. The plan must include a process for rapidly making the decision to decarcerate (delayed decarceration is ineffective with a rapidly spreading pathogen).

Further, that process must include an assessment of the risk associated with an introduction of a pathogen into that specific facility and the harm associated with the specific pathogen for both incarcerated people and staff. Prior discussion must include what level of expected morbidity and mortality would be high enough to trigger emergency decarceration. For example, if decision-makers had assessed that the expected community-level mortality for a population of the size and age of San Quentin would have been eight individuals and that the projected mortality in San Quentin without decarceration was 28 (the subsequently observed mortality), would averting those 20 additional deaths from COVID outweigh the cost and potential dangers of emergency decarceration?

When an outbreak occurs, leadership must take immediate steps to stop transmission in affected housing units, minimize the probability of spread to other housing units and further reduce the risk of rapid spread among groups by reducing maximum occupancy of congregate spaces. This requires planning and implementing three levels of safety: not exceeding safe occupancy under non-epidemic circumstances; emergency reduction of occupancy of high-risk housing units when faced with an epidemic to further reduce transmission risks within the facility; and further emergency reduction of occupancy when an outbreak occurs within an institution (converting affected housing units into safe quarantine and reducing risk in unaffected housing units). This must depend upon a specific pathogen’s transmissibility and lethality.

In March 2020, CDCR prisons averaged 130% design capacity (range: 91%–170%). While addressing overcrowding by decarceration during the COVID-19 pandemic is a recommendation based on public health guidance, implementing this recommendation has been difficult and politically fraught. Between April 2020 and July 2021, CDCR accelerated the release of incarcerated persons, primarily people close to the end of their sentences and a small number of medically vulnerable individuals. These efforts have been documented both before and during the COVID-19 pandemic. The primary driver in occupancy reduction was suspension of intake from local jails.

**Figure 3:**
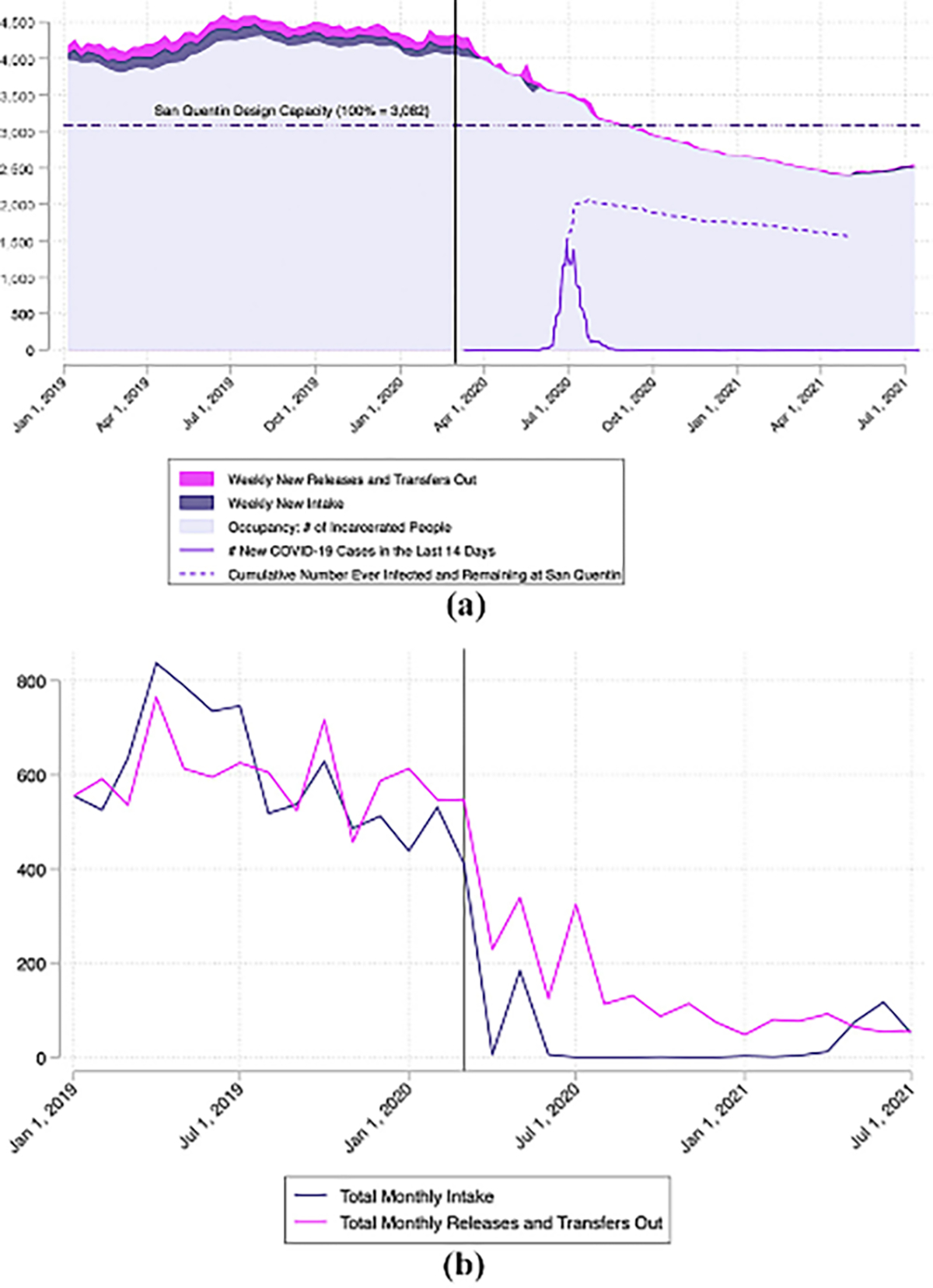
Steady decline of occupancy appears to occur from suspension of intakes from jails and natural/compassionate releases throughout the course of the pandemic (including before, during and after the outbreak), rather than through the need for pandemic-related mass decarceration policies, despite the outbreak affecting a large percentage of San Quentin’s population **Notes:** (a) Occupancy with weekly new releases/transfers out and weekly new intake, design capacity, total new COVID-19 cases in the past 14 days and cumulative number ever infected and remaining at San Quentin; (b) total monthly intake and releases/transfers out at San Quentin between January 1, 2019, and July 17, 2021. Black vertical line represents March 1, 2020. Figure 3(a) depicts the number of new COVID-19 cases within the past 14 days among incarcerated people at San Quentin (solid line), alongside CDCR weekly population report figures for San Quentin’s design capacity of 3,082 (dash-dotted), and number of incarcerated people (left axis) and percent occupancy of design capacity (right axis). On May 5, 2021, San Quentin’s weekly population count reached its lowest during the pandemic at 2,384 incarcerated individuals (77% design capacity), and since then, the number of incarcerated people has been slowly rising – as of October 6, 2021, there are 22,842 incarcerated people (92% design capacity) residing at San Quentin. In short, while San Quentin’s population declined since the onset of the pandemic, occupancy numbers are rising again and the majority of the drop in population can be attributed to keeping people in other carceral settings, such as jails

Figure 3(a) shows San Quentin occupancy declining between April 2020 and April 2021. This reduction was achieved by nearly ceasing intake to the prison. Figure 3(b) shows the absolute number of releases/transfers out of San Quentin, as well as intake over the same period. Release and transfers out averaged 595 per month between January 2019 and February 2020, compared to 150 per month between March and July 2020. Apart from an uptick in releases/transfers out during the peak of the outbreak (28 of which were deaths), there was no appreciable increase in releases following the outbreak. While San Quentin’s population declined because the onset of the pandemic, occupancy numbers are rising again.

Decarceration, a combination of early release with reentry support, furlough (temporary release) and alternative (e.g. home, hotel) confinement, can be considered in an intersectional community effort that is not only effective as a public health intervention, but an integral component of both public safety and community rebuilding. Emergency decarceration measures – alongside reentry planning–should be part of any future prison pandemic preparedness plan.

Approaches to emergency evacuation of high-risk housing units have been reviewed elsewhere (National Academies of Sciences, Engineering and Medicine, 2021). Inside prisons, future research should focus on strategies to effectively use decarceration to mitigate pathogen spread and how to measure and use measures of objective crowding as they relate to ventilation/filtration as well as transmission of infectious pathogens (Simpson *et al.*, 2019; Simpson and Butler, 2020). More research is needed to examine levels of decarceration that would effectively mitigate pathogen spread, for example, expanding on recent work by Towers *et al.* (2022). Further, there remain potential challenges and risks that accompany decarceration as well, which have been discussed in other literature and should be considered when designing these processes (Shepherd and Spivak, 2020). Additional quantitative and qualitative research are needed on how to bolster reentry and post-release support services to reduce the negative externalities that exist for people who are transitioning back to society, which are magnified during a pandemic. We further recognize that it is difficult to isolate the discussion of emergency decarceration from the discussion of reversing the underlying epidemic of mass incarceration in the USA – a moral and public health crisis requiring deep societal reckoning and wholesale policy reform with an importance equivalent to, if not surpassing, that of the ongoing COVID-19 pandemic. As part of those reforms, the USA must also grapple with the extremely long sentences given to young offenders in sharp contrast to our peer countries. As a result of these differences in sentencing, these countries do not incarcerate elderly, disabled persons (at high risk for COVID-related complications) at anywhere close to the rate we do in the USA.

## Conclusion

At this time in the COVID-19 pandemic, decarceration and high levels of vaccine coverage are especially important in large congregate units or areas with shared airspace where ventilation or air filtration cannot be fully achieved, as is the case in many carceral facilities. In the event of a future introduction of an infectious disease that has a higher mortality rate and a lack of effective vaccines, it is clear that prisons should have a plan in place that identifies threat level(s) for when emergency actions (such as decarceration) should be implemented and procedures that enable leadership to make that decision. The lessons from San Quentin prison are instructive for all settings that are infrastructurally incapable of keeping thousands of people safe during a respiratory outbreak. The lessons also speak to the urgency of better preparing for our next uncontrollable outbreak. With over 10 million people incarcerated around the world, 2.1 million of whom live in 5,000 carceral facilities in the USA, further research is urgently needed to inform the most appropriate and the safest levels of multi-layered mitigation strategies, including ventilation and decarceration measures, to protect against respiratory pathogens in these settings.

## Supplementary Material

supplement

## Figures and Tables

**Table 1:** San quentin had greater proportions of people who were older and/or at higher risk for severe outcomes from COVID-19 when compared to the CDCR-wide population

	(1)	(2)	(3)	(4)
	2020 population at CDCR	2020 population at San Quentin	COVID cases at San Quentin	Individuals who died because of COVID at San Quentin
	*N* = 110,859	*N* = 3,857	*N* = 2,241	*N* = 28
Room type				
*Cell*	65.2	84.8	99.5	100.0
*Dorm*	34.8	15.2	0.5	0.0
*Room*	0.01	0.01	0.0	0.0
Aged 55+	18.7 (39.0)	38.7 (48.5)	47.4 (49.9)	92.9 (26.2)
Asthma	13.2 (33.9)	13.7 (34.5)	13.9 (34.4)	25.0 (44.1)
Advanced liver disease	3.3 (17.9)	5.0 (21.9)	5.9 (23.7)	25.0 (44.1)
Morbid obesity	4.0 (19.6)	4.6 (21.4)	4.6 (21.3)	7.4 (26.7)
Cancer	2.8 (16.6)	6.0 (23.8)	7.7 (26.6)	14.3 (35.6)
Chronic obstructive pulmonary disease	2.9 (16.6)	5.5 (22.8)	7.5 (26.2)	32.1 (47.6)
Cardiovascular disease	2.5 (15.6)	4.7 (20.9)	5.5 (22.7)	7.1 (26.2)
Diabetes	8.1 (27.3)	13.0 (33.7)	15.4 (36.1)	50.0 (50.9)
Dialysis	0.1 (8.8)	0.1 (8.4)	0.1 (8.8)	0.0 (0.0)
HIV	0.8 (8.5)	3.0 (17.1)	2.9 (16.8)	0.0 (0.0)
Lung disease	0.1 (3.7)	0.2 (3.9)	0.2 (4.7)	7.1 (26.2)
Immunocompromised	1.3 (11.3)	2.6 (15.7)	2.9 (16.4)	10.7 (31.5)
*Panel (b) CDCR population general medical risk profile and COVID risk scores*				
Available data (of N)	110,859 (100%)	3,857 (100%)	2,236 (99.8%)	28 (100%)
	individuals	individuals	individuals	individuals
General clinical risk *High risk priority 1 level*	1.8 (0.9)	2.1 (1.1)	2.3 (1.1)	2.8 (3.7)
(trigger 2+ high risk selection criteria) *High risk priority 2 level*	8.6%	19.0%	21.0%	17.9%
(trigger 1 high risk selection criterion) *Medium risk level*	6.4%	12.8%	17.2%	53.6%
(trigger at least 1 chronic condition) *Low risk level*	34.5%	31.7%	29.8%	25.0%
(includes subset with well-managed stable conditions)	50.5%	36.5%	32.0%	3.6%
COVID risk score (weighted)	1.3 (2.1)	2.3 (2.7)	2.8 (2.9)	5.0 (0.8)

**Notes:** Panel (a) reports percentages (standard deviations in parentheses) of individuals for the population and population size noted by column. Data source for Column (1): CDCR. Data source for column (2): www.cdcr.ca.gov, as of June 3, 2020. For column (3): CDCR reported the population to be 2,553 on March 19, 2021. Panel (b) reports average general clinical risk (standard deviation in parentheses) with percentage of people at each level, as well as average COVID risk score (standard deviation in parentheses). Appendix B provides detailed descriptions of the scores. General clinical risk level is defined by CDCR. High risk priority 1 is assigned to patients who trigger at least two risk factors. High risk priority 2 is assigned to patients who trigger only one risk factor. Medium risk is assigned to patients with at least one chronic condition who do not meet the criteria for high or low risk. Low risk is assigned to patients who do not meet the selection criteria for high or medium risk categories. A COVID risk score is assigned to each patient at a CDCR institution. The score is a sum of weights associated for different COVID risk scores. A weight score of 4 is assigned for: having age 65 years or above. A weight score of 2 is assigned each for: high risk cancer, COPD, immunocompromised (any of the following conditions: aplastic anemia, histiocytosis, immunosuppressed, organ transplant, other transplant), on dialysis, has advanced liver disease (cirrhosis/end stage liver disease as defined by the CCHCS advanced liver disease condition specifications). A weight score of 1 is assigned each for: active pregnancy, persistent asthma (moderate or severe), chronic lung disease (any of the following: cystic fibrosis, pneumoconiosis, or pulmonary fibrosis), diabetes, high risk diabetes, heart disease (any of the following: cerebrovascular, congestive heart failure, congenital heart disease, ischemic heart disease, peripheral vascular disease, thromboembolic disease, valvular disease), high risk heart disease, HIV/AIDS, poorly controlled HIV/AIDS (HIV with CD4 count <200), morbid obesity (BMI of 40 or above), other chronic conditions. As of July 2020, the following were added. A weight score of 1 assigned to: chronic kidney disease, advanced chronic kidney disease/renal failure (Stage 5 chronic kidney disease or is identified as currently receiving hemodialysis, hemoglobin disorder (separated as its own comorbidity, previously under other chronic conditions), hypertension, neurologic conditions (previously under other chronic conditions), obesity (adjusted to include BMI of 30 or above, previously was 40 or above)

Panel (a) Percentages of people incarcerated in CDCR prisons and people incarcerated at San Quentin across COVID-19 risk factors in 2019–2020
