# Supplementary material for: Respiratory pandemic preparedness learnings from the June 2020 COVID-19 outbreak at San Quentin California State Prison
Source: Int J Prison Health. Author manuscript; Available in PMC 2023 Oct 20. (PMC10231421; doi:10.1108/IJPH-12-2021-0116)
Supplement: Supplementary file 1 [file NIHMS1894029-supplement-supplement.pdf]

Supplemental Appendices

Supplement to:

Kwan, A., Sklar, R., Cameron, D. B., Schell, R., Bertozzi, S. M., McCoy, S. I., Williams, B., Sears, D. A. Respiratory Pandemic Preparedness Learnings from the June 2020 COVID-19 Outbreak at San Quentin California State Prison. In press at IJPH, 2022.

Appendix A: Urgent Memo on the San Quentin COVID-19 Outbreak

Urgent Memo

COVID-19 Outbreak: San Quentin Prison

June 15, 2020

San Quentin California State Prison is experiencing a rapidly evolving COVID-19 (SARS-CoV-2) outbreak with profoundly inadequate resources to keep it from developing into a full-blown local epidemic and health care crisis in the prison and surrounding communities. The urgent resources San Quentin requires range from human capital to environmental risk reduction and rapid testing. Failure to meet these urgent needs will have dire implications for the health of people incarcerated at San Quentin, custody, staff, and the healthcare capacity of Bay Area hospitals. This document provides suggested guidance on immediate actions needed to address the outbreak with emphasis on both the short- and longer-term health of people currently incarcerated at San Quentin.

Background

San Quentin arrives at this tenuous moment with several significant assets including a strong Chief Medical Executive (Dr. Alison Pachynski) and a Chief Physician and Surgeon (Dr. Shanon Garrigan) who have spent the past 3.5 months doing everything in their power to prepare for an unavoidable COVID-19 outbreak. However, these two physicians, even with the enormous assistance they have received from many other healthcare staff, including a strong public health nurse, and a notably excellent partnership with custody leadership (Acting Warden Ronald Broomfield and the recently arrived Health Care Chief Executive Clarence Cryer), is simply not enough to meet the needs at San Quentin. As a result, there are multiple vulnerabilities that we witnessed at San Quentin during our visit on June 13, 2020 which must be urgently addressed to protect the health and safety of the thousands of people incarcerated there as well as staff and surrounding community members.

Although this memo outlines the urgent needs of San Quentin Prison, it is our belief that most – if not all – of these recommendations are important for all California Prisons that are certain to experience an outbreak if they have not already.

Urgent needs and immediate actions required:

1. **Develop a COVID-19 Outbreak Emergency Response Team:** At present, the over-reliance on existing local medical and custody staff to develop an outbreak response plan means that they are tasked with making multiple acute decisions on a daily basis without adequate resources, options, or support to operationalize a centralized plan or long term strategy. This responsibility – overwhelming on its own – is then magnified with the additional necessity of providing implementation oversight of the ad-hoc outbreak plan. Instead, local leadership should have a team of staff who can implement and recommend adjustments to the overarching central COVID-19 control strategy as needed on the local level. There simply do not appear to be sufficient on-the-ground staff who are not working from home. This daily management of the acute phase of the outbreak has the secondary effect of making the lead physicians less available to coordinate the care and treatment of patients incarcerated at San Quentin who become acutely ill in the facility and also increases the vulnerability of San Quentin to errors with potentially dire consequences. Minimum positions required for such a team are included below. Dr. Pachynski and Dr. Garrigan appear to be personally responsible for all of the tasks described below with insufficient tools to support their success. While there may be some central guidance and support offered, additional human capital is urgently needed to achieve the CCHCS's pandemic response goals.

   Minimum Recommended Leadership Team Positions:

   - **Environment of Care Leader.** This position would be responsible for evaluating and addressing immediate needs regarding the physical plant of the prison for ventilation, sanitation, path of patient flow (e.g., developing policies and procedures for how people incarcerated at San Quentin who become infected are transferred through and out of the institution for care) and planning for how to reconfigure and reimagine needed space for quarantine, general population, or medical isolation units depending on how the number of affected people increases or decreases over time. This position would also work with plant operations to ensure that all air vents are cleaned and well functioning and would organize the creation of (a) field hospital(s) or quarantine tents as needed.

   - **Healthcare – Custody Coordination Leader.** This position would focus on coordinating with Custody (and working closely with the Staff Healthcare Liaison Leader, described below) to review current placement on a daily basis, and to determine the appropriate way to cohort people currently incarcerated at San Quentin, staff, and custody including developing quarantine areas (in partnership with the Environment of Care Leader) to minimize risk of infection. This position would also be responsible for ensuring that all transfers *into* San Quentin are halted and that appropriate and timely testing is done to facilitate transfer out of Medical Isolation and Quarantine within the facility, to the community, and – in certain circumstances - to other facilities if medically necessary.

- **COVID-19 Testing Leader.** This position would be responsible for coordinating with the testing center (at this moment, QUEST Diagnostics) including reaching out through public and private sources and coordinating with the state and local departments of public health to improve testing turnaround time, running the list with medical staff (and the Epidemiologist, described below) on a daily basis to determine who has – and who needs – testing, and coordinating contact tracing in response to testing results and reporting of symptoms throughout the facility.

- **Staff Healthcare Liaison Leader.** This position would work with custody leadership (and Union representatives, as appropriate) to cohort staff/custody, develop plans that eradicate staff/custody working within more than one unit in rapid succession, train and enforce PPE rules, support contact tracing and administrative leave needs among exposed and infected staff/custody, and investigate alternatives to potential staff/custody transmission opportunities such as shared vanpools. This position would also track daily staff movements in order to assist with contact tracing when needed.

- **Epidemiologist Analyst Leader.** This position would be responsible for maintenance of a line listing of all active and resolved cases (people incarcerated at San Quentin and staff) and for all data analysis and reporting. This position would also be responsible for a "patient tracking process" of the facility including daily review of the COVID-19 Monitoring Registry to provide close scrutiny of who has tested positive or is in quarantine – where they are currently located (and were recently located), and the same for those who have tested negative. In addition, this position would assist the Environment of Care leader and the Healthcare – Custody Coordination Leader to manage patient movement to quickly clear people when they have tested negative and return them to the General Population and/or to the community. This position would also manage testing data (e.g., in the Reception Area, some have been tested 3-4 times and test results are coming in at different times).

2. **Address Unsafe Overcrowding.** There are currently 3547 people in total incarcerated at San Quentin, approximately ~1400 of whom have at least one COVID-19 risk factor (as do many, unknown, staff members). This means these individuals are at heightened risk of requiring ICU treatment and/or mortality if infected. We detail the units of most immediate concern below. Given the unique architecture and age of San Quentin (built in the mid 1800s and early 1900s), there is exceedingly poor ventilation, extraordinarily close living quarters, and inadequate sanitation. **We therefore recommend that the prison population at San Quentin be reduced to 50% of current capacity (even further reduction would be more beneficial) via decarceration**; this will allow every cell in North and West blocks to be single-room occupancy and would allow leadership at San Quentin to prioritize which units to depopulate further including the high-risk reception center and gymnasium environments. It is important to note that we spoke to a number of incarcerated people who were over the age of 60 and had a matter of weeks left on their sentences. It is inconceivable that they are still in this dangerous environment.

Housing units of most concern at San Quentin at present time:

- **North Block and West Block** have cells with open-grills, and are each 5-tier buildings with a capacity of 800 persons. Ventilation is poor – windows have been welded shut and the fan system does not appear to have been turned on for years; heat on the far side of the building can be stifling. Over 50% of those incarcerated in these units have at least 1 COVID-19 risk factor, and an alarming ~300 have 4 or more COVID-19 risk factors. An outbreak in North and West blocks could easily flood – and overwhelm – San Quentin as well as Bay Area hospitals. (For example, see San Francisco hospital capacity: **https://data.sfgov.org/stories/s/Hospital-Capacity/qtdt-yqr2/**)

- **Reception center** currently has ~500 persons. In the reception Center's "Badger Unit" where people from CIM were transferred, the fear and outrage among the people incarcerated are palpable – people are yelling throughout the housing unit due to discontent about the COVID-19 situation including intake of transfers from CIM and loss of privileges/disruption to daily routine (thereby increasing the risk of COVID-19 spread throughout the tiers via respiratory droplets). It is hard to imagine that as a result of these conditions, that violent incidents will not occur—further threatening the safety and health of the people incarcerated in these units and staff alike.

- **The Gymnasium**, which has been converted to a dorm. There is little to no ventilation in this unit creating high-risk for a catastrophic super spreader event.[1] At a minimum, the gymnasium beds should be spread out more to ensure additional distance between beds, and the second set of doors in the gymnasium dorm must be opened to ensure air turnover. **This unit should be prioritized for closure as a dorm, once sufficient population reduction has been achieved through release.**

- **HVAC – in all units above and in other areas,** there is an immediate need to clean and turn on all fan and HVAC systems immediately (e.g., North Block, Gymnasium, Dorms) in order to maximize air exchange and ventilation as soon as possible. Of note, the exhaust pumps and filters appear dirty on visual inspection, and require clearing and cleaning. Since maximizing air exchange through better ventilation decreases COVID-19 transmission, doors and windows should be opened as much as possible (some have been welded shut and must be remediated). Note that the important aspect is *air exchange*, not the movement of air within the room. Fans that blow air around may help cool people, but they don't decrease rebreathing aerosols unless they filter the air or increase air exchange (diluting the aerosol).

---

[1] It is important to recognize that all of our recommendations regarding ventilation in different housing units at San Quentin were based on the observations of a team of public health professionals accompanying San Quentin medical staff. Although incarcerated persons and custody staff shared their understanding of the ventilation systems in the units and their operability, we neither had the opportunity to speak with any of the facilities staff nor were any members of our team experts in HVAC. We would strongly recommend seeking the advice of such experts and monitoring CO2 levels in different parts of the prison as one easy measure of the extent of rebreathing in a housing unit.

3. **Immediately Improve Testing.** It is inconceivable that in the Bay Area the medical leadership at San Quentin is having to manage an outbreak in their massive antediluvian facilities with PCR tests on a 5-6 day turn-around time. We would argue that there is no higher testing priority for around 100 miles and resources need to be shifted immediately to respond or there will be a massive, uncontrollable outbreak (if it is not too late already). In addition (and this certainly goes without saying), **transfers into San Quentin must be halted immediately**. **Further, priority must be placed on reducing the prison population at San Quentin via decarceration as it will be extremely difficult to ensure the health and safety of all people in this extraordinarily old and complex facility. The following recommendations both support these imperatives and, in some cases, are dependent on their implementation**:

   - **Liaise with testing laboratory to streamline testing,** including exploring observed self-collection of samples and alternate anatomic sites of testing (e.g. saliva, nares swabs).

   - **Improve testing turnaround time at QUEST or go through other laboratories that will be able to improve turnaround time (5-6 days or more is completely unacceptable).** As an example, CMC was able to rapidly respond to their outbreak with a turnaround testing time of 24 hours at some points in the outbreak. Large-scale testing with rapid receipt of results is essential to allow the medical team to minimize community spread. If tests are sent to laboratories other than QUEST, support must be provided to San Quentin to add these results to the EMR as the current process of scanning and manual entry is overly laborious and resulting delays may lead to medical decisions based on outdated data.

   - **The California Department of Public Health** should be compelled to prioritize specimens from San Quentin given the potential for super-spreading in that environment.

   - **Testing of symptomatic patients must be done with individual testing. Testing of asymptomatic patients to identify people who are shedding virus can be done with pools of samples. Without additional information, pools of 10 should be used.** This approach can be used for frequent retesting of people at especially high risk of spreading the virus (staff/custody and people incarcerated in larger units — i.e. almost all of San Quentin).

   - **San Quentin requires on-site testing –** including cartridges and well-trained staff to conduct these (currently they have inadequate staffing to conduct mass swabbing). Sample transport just adds time. San Quentin will need high volume testing for many months, perhaps years. They should have testing capacity on-site and available round-the-clock.

- **Of note, because testing time is so slow, little to no contact tracing can happen. Furthermore, people incarcerated at San Quentin cannot be appropriately transferred within the prison based on test results if results are returned 6 days later and new exposure may have occurred in the interim.** As a result, *entire units are put on lockdown status for the span of a quarantine*. This is not a viable solution. In the long term, as this pandemic will last at least another year and likely longer, this will have profound physical and mental health consequences for the incarcerated population and staff alike.

4. **Develop Additional Medical Isolation and Quarantine Housing.**

Background: It is our understanding that on May 30, transfers from CIM arrived at San Quentin on five buses. Several among those who were transported on Bus 5 tested positive at arrival. While all transfers on Bus 1 and 3 initially tested negative, several later developed COVID-19 symptoms. At the time of our visit, there were no reports of symptoms or positive tests among those who traveled on Buses 2 and 4. At the advice of the local health department, all individuals from the five CIM buses who tested positive or reported symptoms were placed in the Adjustment Center. Those who either tested negative or did not report symptoms were placed individually and in every other cell on the Reception Area's Badger and Donner Units 4th and 5th tiers (among people who were incarcerated at San Quentin prior to the transfer).

June 13 Visit: As of our visit, those requiring *Quarantine* (i.e., people with a credible exposure to COVID-19 who are asymptomatic) are in the Reception Area's Carson Unit. Those requiring *Medical Isolation* (who have tested positive for COVID-19 **or** who have symptoms suggestive of COVID-19 and are still awaiting testing) are in the Adjustment Center as this is the only unit at San Quentin that has single cells with solid doors. Per our notes, there are ~106 cells in the Adjustment Center, with ~80 occupied at the time of our visit.

Urgent Concerns:

1. A massive outbreak at San Quentin will significantly and quickly overwhelm the availability of these 106 Adjustment Center cells, and there will quickly be nowhere for infectious cases to be moved. Further, we cannot emphasize enough the incredible fear that residents we spoke with expressed about being moved to cells typically used for administrative segregation/punishment or "death row" – potentially resulting in short- and long-term mental health consequences. Especially given that early identification of suspected COVID-19 cases depends on reporting of symptoms, **quarantine strategies relying on the Adjustment Center or cells usually used for punishment may thrwart efforts for outbreak containment as people may be reluctant to report their symptoms**. In addition, people with COVID-19 are known to experience rapid physical decompensation; it may therefore be particularly detrimental for a patient with

COVID-19 to be behind a solid door in the most secure areas of the prison out of the sight of medical or nursing staff in the case of an emergency. This may be particularly risky if there are structural barriers to communicating distress to staff (e.g., if accomodations are not readily accessible for people with disabilities or who speak other languages, and/or there are multiple security stages to pass through).

Given San Quentin's antiquated facilities, poor ventilation, and overcrowding, **it is hard to identify any options at San Quentin where it is advisable to house high-risk people with multiple COVID-19 risk factors for serious morbidity or mortality**. Again, for these reasons it will be exceedingly hard for medical staff to keep people safe from contracting COVID-19 at San Quentin and, once infected, it will be very hard to ensure that they do not pass the infection on to others with high health risks or experience rapid health declines themselves. **San Quentin is an extremely dangerous place for an outbreak, everything should be done to decrease the number of people exposed to this environment as quickly as possible.**

Our recommendations for Quarantine and Medical Isolation are as follows:

- Immediately create a field hospital by **converting nearby chapels (there are 3) or even the chow hall.** This field hospital can be designated for all people with confirmed COVID-19 ("Medical Isolation Unit") as there are not substantial risks to isolating infected patients together and these patients would then have access to supervising nurses who could regularly check their respiratory status and comfort levels. Such a unit could have different tiers of medical supervision as some people in medical isolation will be asymptomatic and will not require as close medical supervision. The chapels are large rooms with road access for ambulances and other transport. We recognize the plans for assigning units will become increasingly complex as people of multiple security levels require Quarantine or Medial Isolation. **This again reinforces the need for release** and a dedicated team leader (the **Healthcare – Custody Coordination Leader)** who oversees the work of partnering with custody to identify medically appropriate cohorting solutions.

- **For those currently in the Adjustment Center:** As individuals test negative (via recovery or because they never developed infection) they ideally should be moved out of the Adjustment Center as quickly as possible. However, with evidence of community spread at San Quentin, extreme caution must be exercised when moving persons out of the Adjustment Center who test negative for COVID-19 and who are at high risk for poor health outcomes if infected. For these individuals, we strongly recommend that central

administration work with medical leaders at San Quentin to identify options for safer placement of individuals leaving the Adjustment Center (perhaps in temporary tents) or in other CDCR facilities (transfers would have to happen with exceptional caution given prior failure with transport including 2 weeks of quarantine on either side of transfer coupled with testing at the outset and end of 14-day quarantine in each site). Alternative housing options outside of San Quentin should also be explored, including nearby hotels or school dorms that can be converted in an effort to save lives. People at the Adjustment Center who test positive should be immediately moved to the new Medical Isolation Unit (e.g., in the converted chapels).

- **Physical and mental health during quarantine and medical isolation must be prioritized with adequate consideration for how need may vary across people incarcerated at San Quentin.** While awaiting testing results, people should receive resources to support their well-being as much as possible during isolation/14-day quarantine period (quarantine should not exceed 14 days after a single exposure). Such resources, at a minimum, should include free access to personal tablets with movies, increased access to free canteen items, personal effects and free phone calls, perhaps on state-owned cell phones, and daily opportunities for yard time. While some of these comforts may seem beyond the normal routine of prisons in California, they are simple, low-cost measures that are essential if there is any hope of minimizing the risk of adverse short- and long-term physical and mental health outcomes of isolation among those who are currently in the Adjustment Center for quarantine or isolation. Alternatives for isolation or quarantine that do not involve the Adjustment Center must be immediately sought (e.g., quarantine tents or other areas of the prison where significant depopulation can allow for fewer occupied cells). **Ultimately, there are simply too few options for safe quarantine at San Quentin without prioritizing population reduction through release.**

5. **Improve General Prevention efforts throughout the facility.** In particular, we witnessed alarmingly suboptimal mask use by staff, and three "medical pass nurses" sitting in a work room without masks. Moreover, custody work stations are not set up to physically distance, no additional workstations appear to have been built yet. As a result, even with the best of efforts, officers wind up clustered near each other around a central podium. An infection control nurse and environmental assessment would go a long way towards identifying opportunities to partially alleviate these problems.

6. **Staff Cohorting is a necessity.** At present work shift plans are inadequate from a public health perspective. For example, we learned about staff who were working in the Medical Isolation Unit (Adjustment Center) during the shift and were scheduled to work the next shift in the dorms. This is an enormous risk for the spread of COVID-19 between units.

7. **Convene COVID-19 Inmates Council.** To ensure urgent health messaging is comprehensively communicated through trusted paths, we recommend that a COVID-19 Inmates Council be established (if one does not yet exist) in collaboration with any existing leadership groups/councils among people incarcerated at San Quentin. This council should be asked to provide critical feedback regarding all the above recommendations, how they may best be implemented and messaged to the population, and if there are considerations that have not been addressed that will maximize the urgent and long term health needs associated with this outbreak.

8. **Convene COVID-19 Inmate Family Council.** To ensure urgent health messaging is communicated to the families of people incarcerated at San Quentin, we recommend that a COVID-19 Inmate Family Council be established. This council may also provide critical feedback regarding all the above recommendations, how they may best be implemented, and if there are considerations that have not been addressed that will maximize the urgent and long term health needs associated with this outbreak.

Sandra McCoy, PhD, MPH, *Associate Professor of Epidemiology & Biostatistics, The University of California, Berkeley School of Public Health*

Stefano M. Bertozzi, MD, PhD, *Professor of Health Policy & Management and Dean Emeritus, The University of California, Berkeley School of Public Health*

David Sears, MD, *Assistant Professor of Internal Medicine, Infectious Diseases, The University of California, San Francisco*

Ada Kwan, PhD Candidate, *Division of Health Policy & Management, The University of California, Berkeley School of Public Health*

Catherine Duarte, PhD Candidate, *Division of Epidemiology & Biostatistics, The University of California, Berkeley School of Public Health*

Drew Cameron, PhD Candidate, *Division of Health Policy & Management, The University of California, Berkeley School of Public Health*

Brie Williams, MD, MS, *Professor of Medicine, The University of California, San Francisco, and Director of Amend at UCSF*

Amend at UCSF is a health-focused correctional culture change program led by experts in medicine, infectious diseases, public health, and correctional health and policy that is providing correctional leaders, policymakers, and advocates the evidence-based tools they need to protect the health and dignity of those who live and work in jails and prisons during the COVID-19 pandemic.

The University of California, Berkeley School of Public Health is working on the leading edge of research, educating the public, and mobilizing to serve California's most vulnerable populations during the COVID-19 pandemic.

For more information:

https://amend.us/covid

Appendix B: CDCR risk score definitions

1. **General medical risk score**
    a. **High Risk Priority 1 and Priority 2** is based on high risk selection criteria that include (i) diagnoses/conditions associated with current or future risk for adverse health event, (ii) multiple higher level of care events in past 12 months, (iii) prolonged medical bed stays, (iv) patients on 10 or more medications, (v) two or more high risk specialty consultations in past 6 months, (vi) 65 years or older, (vii) any comorbid medium risk diagnoses/conditions that may increase risks for future adverse health events; Chronic conditions constitute any that do not meet the selection criteria for high risk, including patients enrolled in mental health services delivery system and patients with permanent disabilities (ADA) affecting placement. **High risk priority 1** is assigned to patients who trigger at least two risk factors from the criteria stated. **High risk priority 2** is assigned to patients who trigger only one risk factor from the criteria stated.
    b. **Medium risk** is assigned to patients with at least one chronic condition who do not meet the criteria for high or low risk.
    c. **Low risk** is assigned to patients who do not meet the selection criteria for high or medium risk categories. This includes some patients with medical conditions considered to be well controlled, inactive or otherwise at low risk for adverse health events.
2. **Weighted COVID risk score**. The COVID risk score is a sum of weights assigned to healthcare condition specifications for any given incarcerated person. According to CDCR's data dictionary, "While most risk factors were assigned a base value of one point, some conditions were given increased weight, based on scientific literature available at that time." As of April 2021, weights as defined by CDCR were applied as follows.
    a. A weight score of 4 is assigned for: having age 65 years or above.
    b. A weight score of 2 is assigned each for: high risk cancer, COPD, immunocompromised (any of the following conditions: aplastic anemia, histiocytosis, immunosuppressed, organ transplant, other transplant), on dialysis, has advanced liver disease (cirrhosis/end stage liver disease as defined by the CCHCS advanced liver disease condition specifications).
    c. A weight score of 1 is assigned each for: active pregnancy, persistent asthma (moderate or severe), chronic lung disease (any of the following: cystic fibrosis, pneumoconiosis, or pulmonary fibrosis), diabetes, high risk diabetes, heart disease (any of the following: cerebrovascular, congestive heart failure, congenital heart disease, ischemic heart disease, peripheral vascular disease, thromboembolic disease, valvular disease), high risk heart disease, HIV/AIDS, poorly controlled HIV/AIDS (HIV with CD4 count <200), morbid obesity (BMI of 40 or above), other chronic conditions.

    As of July 2020, the following were added.
    (a) A weight score of 1 assigned to: chronic kidney disease, advanced chronic kidney disease / renal failure (stage 5 chronic kidney disease or is identified as currently receiving hemodialysis, hemoglobin disorder (separated as its own comorbidity, previously under other chronic conditions), hypertension, neurologic conditions (previously under other chronic conditions), obesity (adjusted to include BMI of 30 or above, previously was 40 or above).

References

California Correctional Health Care Services. CCHCS clinical risk scoring. Available from: https://cdcrdata.miraheze.org/.

California Correctional Health Care Services. CCHCS criteria for COVID related severe morbidity & mortality: Based on literature reviewed as of 3/29/2020 and discussions with experts. Available from: https://cdcrdata.miraheze.org/.

California Correctional Health Care Services. CCHCS criteria for COVID related severe morbidity & mortality: Based on literature reviewed as of 7/26/2020 and discussions with experts. Available from: https://cdcrdata.miraheze.org/.
